# The Role of Osteoblasts in Phenotypic Variability of Dominant Osteogenesis Imperfecta: Evidence from Patients and Murine Models

**DOI:** 10.3390/ijms262311722

**Published:** 2025-12-03

**Authors:** Milena Jovanovic, Apratim Mitra, Chris Stephan, Ka Wai Wong, Sara Talvacchio, Antonella Forlino, Michael To, Kenneth M. Kozloff, Ryan K. Dale, Joan C. Marini

**Affiliations:** 1 Section on Heritable Disorders of Bone and Extracellular Matrix, Eunice Kennedy Shriver National Institute of Child Health and Human Development, National Institutes of Health, Bethesda, MD 20892, USA; 2 Bioinformatics and Scientific Programming Core, Eunice Kennedy Shriver National Institute of Child Health and Human Development, National Institutes of Health, Bethesda, MD 20892, USA; 3 Department of Orthopaedic Surgery, University of Michigan, Ann Arbor, MI 48109, USA; 4 Department of Orthopaedics and Traumatology, The University of Hong Kong-Shenzhen Hospital (HKU-SZH), Shenzhen 518053, China; 5 Department of Orthopaedics and Traumatology, Li Ka Shing Faculty of Medicine, The University of Hong Kong, Hong Kong, China; 6 Office of the Clinical Director, Eunice Kennedy Shriver National Institute of Child Health and Human Development, National Institutes of Health, Bethesda, MD 20892, USA; 7 Biochemistry Unit, Department of Molecular Medicine, University of Pavia, 27100 Pavia, Italy; antonella.forlino@unipv.it

**Keywords:** Osteogenesis Imperfecta, osteoblast differentiation, phenotypic variability, bone mineralization, mitochondria

## Abstract

One of the hallmarks of Osteogenesis Imperfecta (OI) is phenotypic variability among individuals with the same mutation. The aim of our study is to investigate the under-explored role of osteoblast differentiation in OI phenotypic variability by using human and murine OI osteoblasts. This is the first comparative study of osteoblasts from OI patients vs. healthy pediatric controls. We investigated osteoblasts carrying *COL1A1* substitutions Gly352Ser and Gly589Ser, each expressed in two unrelated patients differing in phenotypic severity. Osteoblasts from type III OI patients with both mutations deposited significantly less mineral vs. type IV. RNA-Seq showed osteoblasts from type IV OI patients with different mutations had downregulated mitochondrial pathways, while osteoblasts from type III OI patients showed downregulation of extracellular matrix pathways. Puromycin assay demonstrated osteoblast protein synthesis was significantly upregulated in type III vs. type IV OI patients. UPR PERK and BiP were reduced in osteoblasts with Gly352Ser from type III and IV OI patients and in osteoblasts with Gly589Ser from a type III OI patient, while both proteins were increased in Gly589Ser osteoblasts from the type IV patient. Additionally, in a murine comparative study, *Col1a1 Gly349Ser*, called Brtl Ser, showed a much more severe skeletal phenotype than Brtl Cys. Brtl Ser calvarial osteoblasts had reduced collagen secretion and folding with abnormal dermal collagen fibrils vs. wildtype. Also, Brtl Ser osteoblasts showed condensed actin filaments but a similar mineral deposition as Brtl Cys. Electron microscopy revealed elongated mitochondria with cristae dropout in patient and mutant murine osteoblasts. Our study yielded novel insights highlighting osteoblast differentiation, mineralization, and a potential role of mitochondria in OI pathology and phenotypic variability.

## 1. Introduction

Osteogenesis Imperfecta (OI) is a genetically and phenotypically heterogeneous bone dysplasia characterized by bone fractures, short stature, and skeletal defects, with secondary effects on other connective tissues such as lung and heart tissue, joints, and teeth [1,2,3,4].

The phenotype of OI caused by defects in type I collagen ranges from moderately severe to perinatal lethal, raising obstacles in understanding OI’s molecular, cellular, and bone tissue mechanisms. One of the hallmarks of OI and other dominantly inherited skeletal disorders is phenotypic variability, the phenomenon of patients having the same OI-causing mutation but exhibiting substantially different levels of clinical severity. The Consortium for OI Mutations reported the genotype–phenotype correlations of 832 mutations located in the helical domain of type I collagen [5]. In this study, around one-third of mutations with glycine substitutions in the α1(I) chain were shown to be lethal, specifically those located in major ligand-binding regions (MLBRs) of collagen, which are important for extracellular matrix interactions. The α1(I) chain had almost three dozen glycine residues, in which both lethal and nonlethal substitutions were identified. In 11 of those cases, the same substituting residue was associated with different phenotypes. For the α2(I) chain, there were eight glycine residues at which both lethal and nonlethal outcomes were found [5].

A more recent analysis of genotype–phenotype correlations in 3152 OI patients with collagen mutations reported extensive phenotypic variability. Glycine substitutions were reported at 217 out of 338 helical glycines in the α1(I) chain. Of these, 97 glycine residues had at least two patients reported with phenotypic variability; variability at 39 glycines was related to variability among non-lethal types, while variability at 58 glycines involved at least one lethal case and one non-lethal case. The data for the α2(I) chain is similar to α1(I) variability, with substitutions reported at 222 of 338 helical glycines. Of those 222 residues, 112 glycine residues had at least two patients reported with phenotypic variability, among which identical substitutions at 63 glycines were related to variability among non-lethal OI types, while identical substitutions at 49 glycines had at least one lethal and one non-lethal OI type. Given the identical collagen mutations, the potential contributions to this variability have been considered to include extrinsic factors such as the non-collagen components of the extracellular matrix and variation in the expression of osteoblast intracellular pathways, including post-translational modifications, UPR, autophagy, mineralization, and cytoskeletal functions [6].

Our study involved an intensive investigation of signaling pathways and modifying factors using human and murine OI osteoblasts to obtain a deeper understanding of how collagen mutations affect osteoblast cellular function. The primary objective of the study is to investigate the role of osteoblast differentiation in phenotypic variability of OI by using human and murine OI osteoblasts with different levels of phenotype severity. We examined patient osteoblasts with substitutions at helical G352S (p.G530S) and G589S (p.G767S) in the *COL1A1* gene. For each mutation, we compared the differentiation of osteoblasts from two unrelated patients, one with OI type III (severe progressive deforming OI) paired with another patient with OI type IV (moderate-severity OI), respectively, to age-matched controls. We also examined murine OI osteoblasts carrying different substitutions at the same α1(I) glycine position to assess their impact on biochemical, cellular, and skeletal phenotypes. Specifically, we compared a new model, Brtl Ser (α1(I) G349S, p.G527S), with our established type IV OI model, Brtl Cys (α1(I) G349C, p.G527C), and delineated their markedly different phenotypic outcomes.

## 2. Results

### 2.1. Osteoblast Signaling Pathways Altered in Classical OI and Potential for Involvement in Phenotypic Severity

To investigate the phenotypic variability of OI, we utilized primary osteoblasts isolated from patients with *COL1A1* point mutations responsible for the G352S and G589S substitutions. For each glycine substitution, we compared two unrelated patients differing in phenotypic severity. We investigated which signaling pathways and modifying factors are altered in osteoblasts from classical OI patients during differentiation by differentiating osteoblasts from two patient pairs, each pair comprising a type III and a type IV OI patient with the same mutation, and unaffected control osteoblasts. See clinical case reports and radiographs in Supplementary Clinical Information. Lysates were collected weekly for 3 weeks and utilized for RNA-Seq analysis.

**Figure 1:**
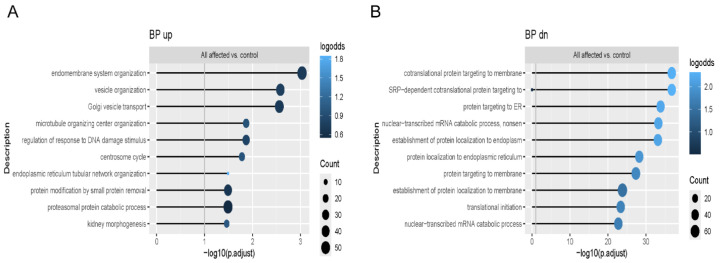
RNA-Seq signaling pathways in human dominant OI osteoblasts. Functional enrichment analysis showing enriched Gene Ontology (GO) terms in the Biological Process ontology, including (**A**) upregulated genes and (**B**) downregulated genes, in comparison of all four human osteoblasts to control osteoblasts. Color indicates a fraction of upregulated/downregulated genes annotated with the term.

RNA-Seq showed upregulation in genes related to vesicle organization, Golgi vesicle transport, and proteasomal protein degradation when the full group of OI patient osteoblasts was compared to controls (Figure 1A). Interestingly, genes downregulated in patient osteoblasts were enriched in several pathways related to protein translation (Figure 1B).

**Figure 2:**
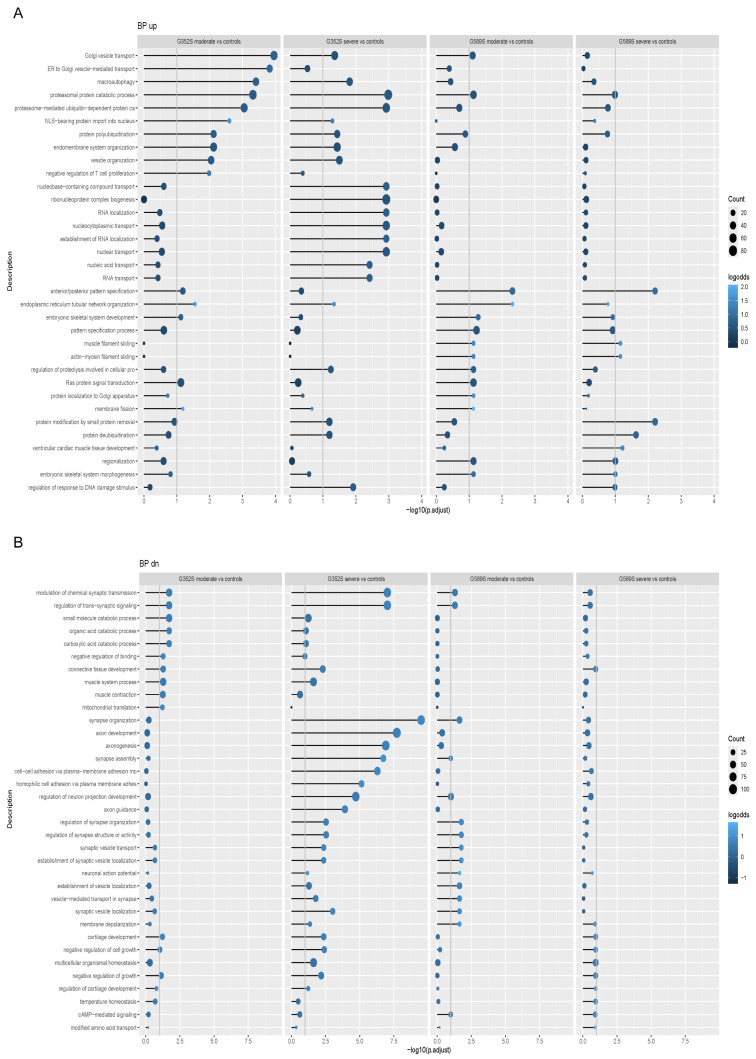
RNA-Seq signaling pathways in human dominant OI osteoblasts. Functional enrichment analysis showing the enriched Gene Ontology (GO) terms in the Biological Process ontology within (**A**) upregulated genes and (**B**) downregulated genes in osteoblasts with the G352S substitution from the type IV OI patient, osteoblasts with the G352S substitution from the type III OI patient, osteoblasts with the G589S substitution from the type IV OI patient, and osteoblasts with the G589S substitution from the type III OI patient compared to control osteoblasts. Color indicates a fraction of upregulated/downregulated genes annotated with the term.

**Figure 3:**
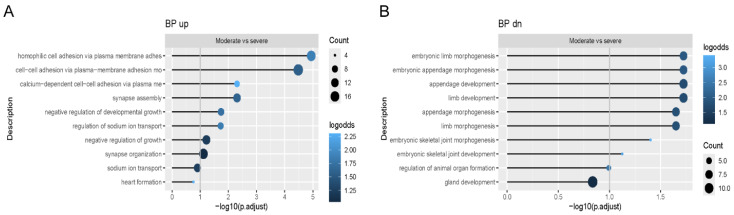
RNA-Seq signaling pathways in human dominant OI osteoblasts. Functional enrichment analysis showing the enriched Gene Ontology (GO) terms in the Biological Process ontology within (**A**) upregulated and (**B**) downregulated genes in comparison of osteoblasts from both type IV OI (G352S, G589S) to osteoblasts from both type III OI (G352S, G589S) patients. Color indicates a fraction of upregulated/downregulated genes annotated with the term.

When we compared osteoblast differentiation from each patient individually to controls, we found that proteasomal protein degradation and ubiquitination were among the most significantly enriched pathways with upregulated genes in patient osteoblasts (Figure 2A), while connective tissue development, mitochondrial translation, and adhesion were among the pathways where genes were downregulated (Figure 2B). In addition, when the two patients with moderate type IV OI were compared to the two patients with severe progressive deforming type III OI, functional enrichment analyses showed enrichment of pathways with upregulated genes governing cell adhesion and negative regulation of developmental growth (Figure 3A). On the other hand, the genes downregulated in moderate vs. severe osteoblasts were enriched in pathways related to embryonic limb and joint development (Figure 3B).

### 2.2. Osteoblast In Vitro Mineralization Correlates with Severity of OI Phenotype

**Figure 4:**
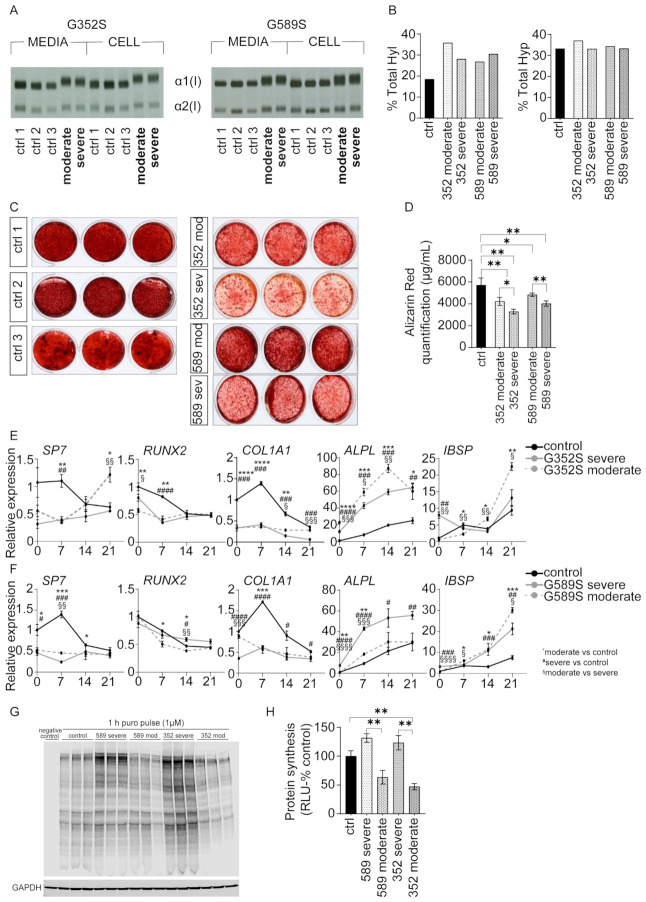
Altered mineralization and protein synthesis in osteoblasts from dominant human OI. (**A**) Type I collagen from media and cell layer of human control and dominant OI osteoblasts was electrophoresed on SDS-Urea-PAGE. (**B**) Quantification of hydroxylysine and hydroxyproline content of secreted type I collagen in control and human OI osteoblasts assessed by HPLC. (**C**) Alizarin Red staining of 6-week-differentiated control and OI osteoblasts in technical triplicates. (**D**) Quantification of Alizarin Red staining by hydrochloric acid extraction. mRNA expression levels of osteoblast marker genes *SP7*, *RUNX2*, *COL1A1*, *ALPL,* and *IBSP* in control and OI osteoblasts from (**E**) G352S patient pair and (**F**) G589S patient pair at Day 0, 7, 14, and 21 of differentiation by RT-qPCR analysis. Expression was normalized to control mRNA expression at Day 0 of osteoblasts differentiation and evaluated by ^ΔΔ^Ct method. (**G**) Western blot of neosynthesized proteins labeled with puromycin in control and human OI osteoblasts. Negative control represents control cells that were not labeled with puromycin. GAPDH was used as a loading control. (**H**) Quantification of puromycin-labeled neosynthesized proteins expressed as a percentage of the values obtained in the control group (RLU-relative light units). Data are presented as means ± SEM. * (^#^ or ^§^) *p* < 0.05, ** (^##^ or ^§§^) *p* < 0.01, *** (^###^ or ^§§§^) *p* < 0.001, **** (^####^ or ^§§§§^) *p* < 0.0001. Comparisons: * moderate to control, # severe to control, § moderate to severe.

To further investigate the phenotypic variability of OI, we first determined the overmodification of intracellular and secreted collagen chains by gel migration of steady-state collagen. Collagen from patient osteoblasts has slower and broader migration of α1(I) and α2(I) chains compared to collagen synthesized by control osteoblasts (Figure 4A). Amino acid analysis of the hydroxylysine and hydroxyproline content of secreted type I collagen revealed the expected increase in hydroxylation of lysine residues in collagen synthesized by all four patient osteoblasts (average 30.3% vs. 18.5% control), but normal hydroxylation of proline residues compared to control. The collagen hydroxylysine content did not correlate with patients’ phenotypic severity, suggesting that extent of overmodification of secreted collagen chains is not a differentiating factor in OI phenotypic severity (Figure 4B).

We next evaluated the mineralization capacity of control and patient osteoblasts in vitro by Alizarin Red staining. All patient osteoblasts deposited less mineral than controls. Interestingly, osteoblasts from patients with type IV OI caused by both G352S and G589S substitutions deposited significantly more mineral than osteoblasts from patients with severe type III OI, indicating that a mineralization defect is a potential aspect of phenotypic variability of the OI phenotype (Figure 4C,D).

Expression levels of osteoblast marker genes during differentiation do not consistently correlate with the distinction in mineralization between osteoblasts from patients with different phenotype severity. Overall, expression of early markers *SP7*, *RUNX2*, and *COL1A1* in osteoblasts from each patient pair is predominantly reduced relative to controls, while later markers such as *ALPL* and *IBSP* show increased expression in patients versus controls (Figure 4E,F). However, distinctions between cells from patients with different phenotypic severity are inconsistent.

Expression levels of early osteoblast marker genes *SP7*, *RUNX2*, and *COL1A1* were reduced in osteoblasts from both moderate and severe patients with a G352S substitution compared to controls, except for significantly increased *SP7* expression on Day 21 in the osteoblasts from the type IV OI patient. Expression levels of later marker genes *ALPL* and *IBSP* were significantly higher in osteoblasts from both patients with G352S substitutions compared to control throughout differentiation. When comparing expression levels between phenotypes, *SP7* was significantly increased on Day 21 in osteoblasts from the type IV OI patient compared to type III with a G352S substitution. *RUNX2* expression was significantly reduced on Day 0 in osteoblasts from the type IV OI patient compared to type III. *COL1A1* expression was significantly increased in osteoblasts from the respective type IV OI patient vs. the more severe type III patient at Day 14 (*p* = 0.015) and 21 (*p* = 0.0001) for the patient pair with G352S substitutions. Osteoblasts from the patient with moderate OI severity had significantly higher *ALPL* transcripts than osteoblasts from the paired patient with the severe phenotype at Days 0, 7, and 14. Osteoblasts from the type IV OI patient with G352S substitution had significantly lower *IBSP* compared to osteoblasts from the more severe paired patient at Day 0 and 7 but significantly increased levels at Day 14 and 21 (Figure 4E,F).

Similarly, osteoblasts from the patient pair with a G589S substitution had significantly reduced levels of *SP7*, *RUNX2*, and *COL1A1* expression compared to the control, except for significantly increased *RUNX2* expression on Day 14 in osteoblasts from the type III OI patient. On other hand, *ALPL* and *IBSP* had increased expression levels. Comparing phenotypes, *SP7* was significantly increased on Day 7 in osteoblasts from the type III OI patient compared to cells from the patient with moderate severity, while *RUNX2* expression was significantly reduced on Day 14 in the osteoblasts from the type IV OI patient compared to type III. *COL1A1* expression was significantly increased in Day 0 (*p* = 0.0003) in osteoblasts with moderate phenotype vs. severe. Expression levels of *ALPL* were significantly reduced on Day 0 and 7 in osteoblasts from the patient with moderate OI severity compared to osteoblasts from the paired patient with the severe phenotype. A significant increase in late expression of *IBSP* was seen in osteoblasts from the type IV OI G589S patient on Day 7 and 21 compared to paired osteoblasts from the severe patient (Figure 4E,F).

### 2.3. Osteoblast In Vitro Protein Synthesis Defect Correlates with Severity of OI Phenotype

In light of the differences in expression of marker genes in osteoblasts from OI patients, both between phenotypes and between early and later markers versus control osteoblasts, we examined total protein synthesis in patient osteoblasts and controls by labeling the cells with puromycin for detection of neosynthesized proteins. Western blots showed reduced amounts of neosynthesized proteins in moderate Type IV OI patient osteoblasts compared to osteoblasts from severe type III OI and control, while type III OI patient osteoblasts trended towards increased protein neosynthesis compared to control (Figure 4G,H). The significantly greater protein accumulation in type III vs. type IV OI correlates inversely with a greater capacity for mineral deposition in vitro of type IV vs. type III osteoblasts, suggesting that increased intracellular protein retention in type III OI may be a contributing factor to mineralization impairment.

### 2.4. UPR Signaling Pathway in OI Phenotypic Severity

**Figure 5:**
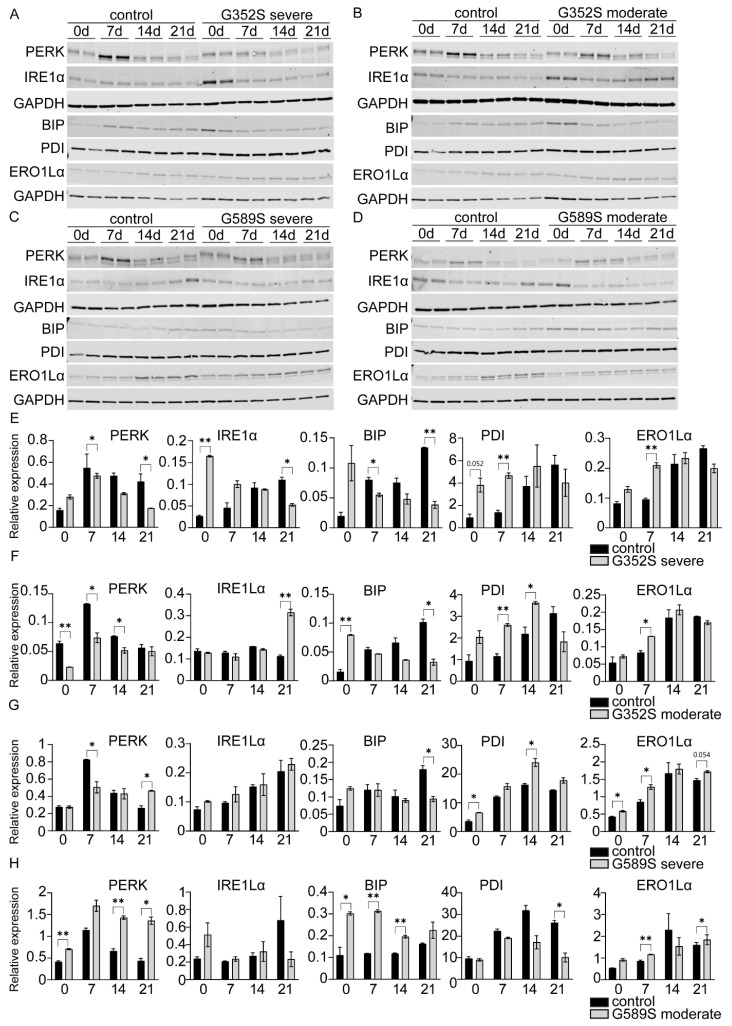
Altered UPR signaling pathway in osteoblasts from dominant human OI. (**A**) Western blots of PERK, IRE1α, BIP, PDI, and ERO1Lα in control osteoblasts and (**A**) osteoblasts with the G352S substitution from the type III OI patient, (**B**) osteoblasts with the G352S substitution from the type IV OI patient, (**C**) osteoblasts with the G589S substitution from the type III OI patient, and (**D**) osteoblasts with the G589S substitution from the type IV OI patient at Day 0, 7, 14, and 21 of differentiation followed by (**E**–**H**) quantification of corresponding protein levels normalized to GAPDH. GAPDH was used as a loading control. Data are presented as means ± SEM. * *p* < 0.05, ** *p* < 0.01.

The unfolded protein response (UPR) signaling pathway, activated by misfolded protein accumulation in the ER, has been shown to be involved in the pathophysiology of dominant and recessive types of OI [7,8]. Therefore, we further investigated the extent to which the UPR pathway is a differentiating factor in the phenotypic variability of OI (Figure 5A–H). PERK protein, one of the main UPR branches, was assayed in patient osteoblasts at four timepoints throughout osteoblast differentiation. The PERK pathway was generally reduced in OI osteoblasts, while in contrast, osteoblasts with the G589S substitution from the type IV OI patient had consistent activation of PERK.

From another UPR branch, IRE1α was significantly increased at baseline in osteoblasts from type III OI with a G352S substitution but significantly reduced at Day 21, whereas IRE1α protein expression was significantly increased at Day 21 with no differences from control on earlier days in the paired osteoblasts from the type IV OI patient. In osteoblasts with a G589S substitution, IRE1α was not significantly different than the control but trended towards increasing throughout the differentiation, except for the Day 21 sample from the type IV OI patient.

BIP, a direct ER stress sensor, activates UPR signaling pathway by dissociating from PERK and IRE1α. For osteoblasts from three patients, both cases with G352S substitutions and the type III OI with a G589S substitution, BIP protein was increased at Day 0 compared to control but then decreased throughout differentiation. Interestingly, in osteoblasts with a G589S substitution from a type IV OI patient, BIP was significantly increased compared to control (Days 0, 7, 14), which coordinates with the consistently increased PERK activation in this patient’s osteoblasts.

PDI, a protein disulfide isomerase, together with ERO1, an oxidative enzyme of PDI, catalyzes disulfide bonds in collagen molecules to facilitate the folding of collagen; they are also involved in UPR signaling [9]. PDI protein levels were mostly upregulated in patient osteoblasts throughout differentiation, except for osteoblasts from the type IV OI patient with a G589S collagen substitution, which had reduced PDI compared to the control (Figure 5G,H). ERO1Lα protein expression was generally increased in all patient osteoblasts compared to the control.

**Figure 6:**
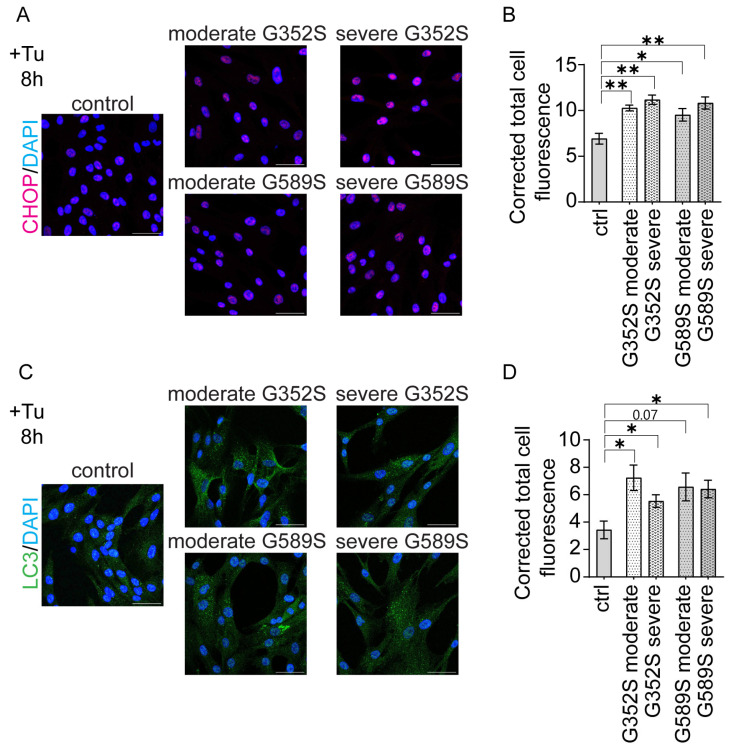
Increased apoptosis and autophagy in osteoblasts from dominant OI. (**A**) Immunocytochemistry and quantification of (**A**,**B**) CHOP and (**C**,**D**) LC3 proteins in control and OI human osteoblasts after 8 h treatment with Tunicamycin. DAPI was used as a counterstain. Scale bars, 50 µm. Data are presented as means ± SEM. * *p* < 0.05, ** *p* < 0.01.

We further examined CHOP and LC3 protein levels by immunocytochemistry staining in patient osteoblasts treated with Tunicamycin for 8 h. The CHOP signal was significantly increased in all patient osteoblasts compared to control. Further, CHOP levels were modestly increased in osteoblasts from type III vs. type IV OI patients (*p* = 0.16 for G352S pair, *p* = 0.19 for G589S pair) (Figure 6A,B). LC3, an autophagy marker, was significantly increased in osteoblasts of three OI patients, with osteoblasts from type IV OI with a G589S collagen substitution trending increased (*p* = 0.07). Also, between OI types, there was a modest trend toward increase (*p* = 0.14) in LC3 in type IV patient osteoblasts compared to type III for the G352S pair (Figure 6C,D).

### 2.5. Comparison of Brtl Cys Murine Model to a New Brtl Ser Mouse Phenotype

**Figure 7:**
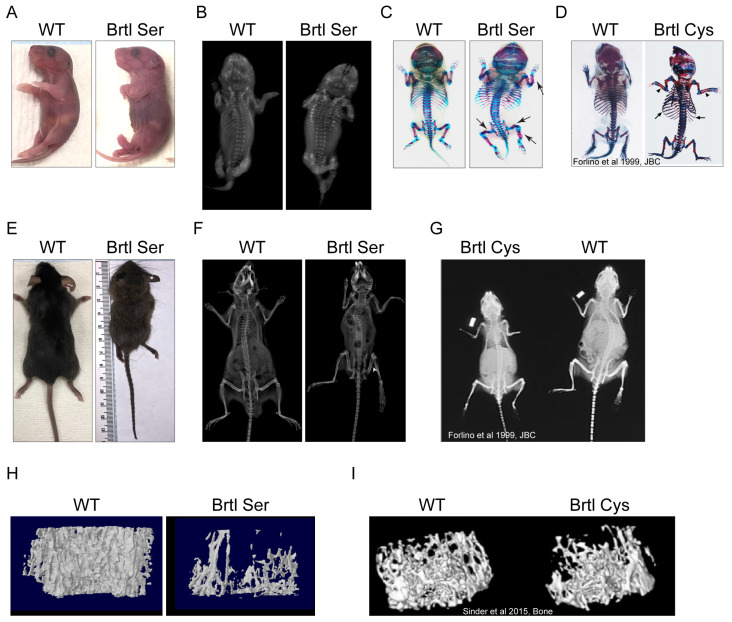
New Brtl Ser murine model exhibits a more severe phenotype compared to Brtl Cys. (**A**) Photography of P1 WT and Brtl Ser pups. (**B**) X-ray images of P1 WT and Brtl Ser pups. Whole-mount skeletal staining of (**C**) Brtl Ser P1 pup and its WT littermate (black arrows pointing to fractures) and (**D**) Brtl Cys P1 pup and its WT littermate (previously published [10]). (**E**) Photography of 7-week-old WT and Brtl Ser mice. X-ray images of (**F**) 7-week-old Brtl Ser mouse and its WT littermate (white arrowhead pointing to a fracture), and (**G**) adult Brtl Cys mouse and its WT littermate (previously published [10]). MicroCT images of (**H**) 7-week-old Brtl Ser femur and its WT littermate and (**I**) 8-week-old Brtl Cys mouse and its WT littermate (previously published [11]).

For studies of phenotypic variability in OI based on different substitutions at the same collagen glycine residue, a new conditional OI mouse model (*Col1a1* Gly349Ser, p.Gly527Ser, henceforth “Brtl Ser”) was generated based on our previously established Brtl Cys (Gly349Cys) mouse [10]. Our goal was to explore the effect of a different helical collagen glycine substituting residue on murine OI skeletal phenotype. Brtl Ser, bred on a C57Bl/6J background with EIIa-Cre activation, is perinatal lethal. However, when the mice were crossed into our CD1-Brtl mixed background [10], several mutants with short-term survival were obtained after EIIa-Cre activation, with 9 weeks as the longest mutant lifespan. Brtl Ser pups have the same length and weight as their wildtype littermates, while 7- and 9-week-old survivors were 1/3 the length and 3/4 the weight of wildtype animals. Brtl Ser had a more severe skeletal phenotype than Brtl Cys mice, with severe kyphosis, bowed limbs, rib fractures, flared ribcage, and poor skeletal mineralization, as shown by X-ray and whole mount skeletal staining of Day 1 newborn pups (Figure 7A–D). Radiographs of 7-week-old Brtl Ser mice showed multiple fractures, which are not seen in Brtl Cys adults. They also had kyphosis and a flared ribcage, similar to Brtl Cys adult mice (Figure 7E–G). MicroCT analysis of femora of 7-week-old Brtl Ser mice revealed significantly reduced bone mass in both trabecular and cortical bone compartments compared to wildtype and Brtl Cys femurs (Figure 7H,I, Table 1).

**Figure 8:**
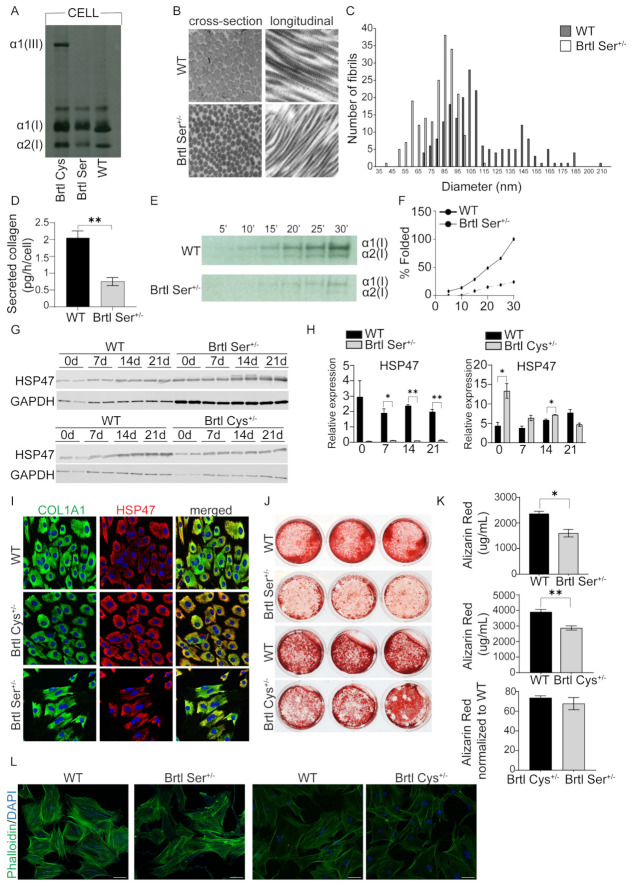
Comparison of molecular changes between Brtl Ser and Brtl Cys osteoblasts. (**A**) Type I collagen from cell layer of WT, Brtl Cys, and Brtl Ser calvarial osteoblasts was electrophoresed on 5% SDS-Urea-PAGE. (**B**) Cross-sections and longitudinal sections of dermal fibrils in 7-week-old Brtl Ser and its WT littermate. Scale bars, 0.2 µm. (**C**) Quantification of dermal fibrils. (**D**) Quantification of secreted collagen from 24 h collected media from Brtl Ser and WT calvarial osteoblasts. (**E**) Intracellular folding of type I collagen in calvarial Brtl Ser and WT osteoblasts. (**F**) Quantification of intracellular folded type I collagen. (**G**) Western blots of HSP47 protein in Brtl Ser and Brtl Cys calvarial osteoblasts compared to WT calvarial osteoblasts at Day 0, 7, 14, and 21 of differentiation followed by (**H**) quantification of corresponding protein levels normalized to GAPDH. GAPDH was used as a loading control. (**I**) Immunocytochemistry of α1(I) collagen (green) and HSP47 (red) in WT, Brtl Cys, and Brtl Ser calvarial osteoblasts. DAPI was used as a counterstain. (**J**) Alizarin Red staining of calvarial osteoblasts in technical triplicates from Brtl Ser and Brtl Cys mice compared to corresponding WT. (**K**) Quantification of Alizarin Red staining by hydrochloric acid extraction of Brtl Ser and Brtl Cys calvarial osteoblasts compared to WT, as well as normalized quantification of each mutant to its corresponding WT. (**L**) Phalloidin staining (green) of actin filaments in Brtl Ser and Brtl Cys calvarial osteoblasts and its corresponding WT. DAPI was used for nuclei staining. Scale bars, 50 µm. Data are presented as means ± SEM. * *p* < 0.05, ** *p* < 0.01.

To further investigate the effect of the G349S mutation on murine bone phenotype, we measured parameters of skin and bone collagen, as well as osteoblast collagen. We determined the overmodification of intracellular collagen chains by gel migration of steady-state collagen. Collagen from both Brtl Cys and Brtl Ser calvarial osteoblasts has slower and broader migration of α1(I) and α2(I) chains compared to wildtype collagen. On other hand, when comparing between mutant collagens, Brtl Ser showed slower migration of both collagen α1(I) and α2(I) chains compared to Brtl Cys (Figure 8A). Brtl Ser dermal collagen fibrils had a smaller cross-section and tighter packing compared to wildtype (Figure 8B,C). Brtl Ser calvarial osteoblasts secreted significantly less collagen in vitro in a 24 hr media collection and displayed slower folding of α1 and α2 type I collagen chains into the collagen trimer (Figure 8D–F).

In previous studies, Brtl Cys murine skin fibroblasts were reported to have swollen ER and induced ER stress due to collagen retention [13,14]. Therefore, we investigated whether HSP47, a collagen specific marker, was altered in Brtl Ser and Brtl Cys calvarial osteoblasts. Brtl Ser osteoblasts had significantly reduced HSP47 protein levels across the differentiation timepoints (Day 7, 14 and 21) compared to WT (Figure 8G,H). In contrast, Brtl Cys osteoblasts had generally increased HSP47 protein expression until late differentiation, with significant increases at Days 0 and 14 compared to WT (Figure 8G,H). Immunocytochemistry staining of wildtype, Brtl Ser, and Brtl Cys calvarial osteoblasts at baseline showed colocalization of HSP47 and type I collagen in the ER; the most distinct co-localization was in Brtl Cys osteoblasts (Figure 8I), complementing the Western results.

We further investigated the in vitro mineralization capacity of Brtl Ser and Brtl Cys calvarial osteoblasts. Both mutant osteoblasts deposited significantly less mineral compared to WT (Figure 8J,K). After normalizing each mutant to its own WT, there was no significant difference in mineral deposition between Brtl Ser and Brtl Cys osteoblasts (Figure 8K). These alterations in mineralization could be impacted by cytoskeletal changes in osteoblasts. Phalloidin immunofluorescence staining showed that Brtl Ser calvarial osteoblasts had condensed actin filaments compared to WT and Brtl Cys (Figure 8L).

### 2.6. Mitochondrial Defect in Human and Murine OI Osteoblasts

**Figure 9:**
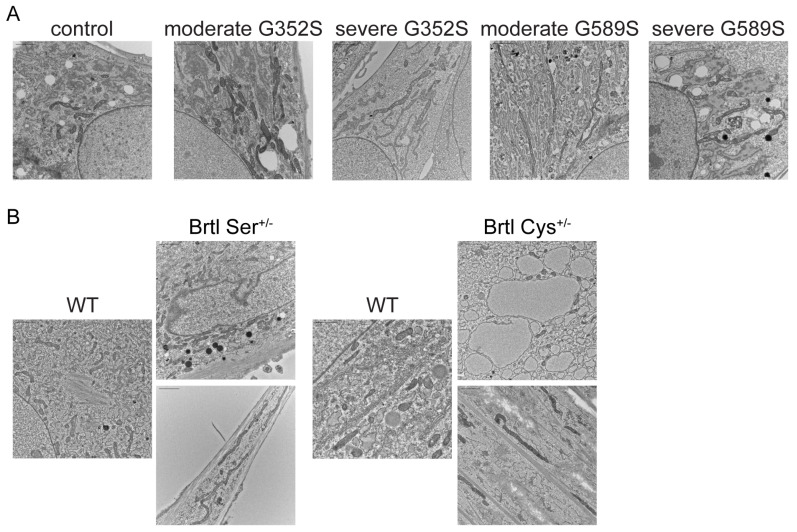
Altered mitochondrial morphology in human and murine OI osteoblasts. (**A**) Electron microscopy images of control and OI human osteoblasts. (**B**) Electron microscopy images of Brtl Ser and Brtl Cys calvarial osteoblasts compared to its corresponding WT. Scale bars, 2 µm.

Recent studies by our group [15] showed for the first time that recessive OI osteoblasts exhibited changes in mitochondrial function in terms of fusion and fission processes that affected the morphology of mitochondria and superoxide production by mitochondria. Due to possible involvement of ER stress in phenotypic variability (Figure 5 and Figure 8), we investigated whether ER stress affected mitochondrial function in patient and murine osteoblasts. Electron microscopy showed elongated mitochondria with evident cristolysis in patient osteoblasts compared to control (Figure 9A). Brtl Ser and Brtl Cys osteoblasts also displayed elongated mitochondria, as seen in human osteoblasts (Figure 9B). These data indicate that mitochondrial dysfunction may be a common cellular feature of OI.

## 3. Discussion

In this combined study of OI human and murine osteoblasts, we aimed to uncover the underlying mechanisms and modifying factors that play a role in phenotypic variability, a hallmark of OI and many other skeletal dysplasias, by comparing two pairs of patients with the same mutation but different degrees of severity (type III vs. type IV OI), as well as comparing bone phenotypes of OI murine models with a different substitution at the same α1(I) helical glycine residue.

We show that osteoblasts from patients with a moderate OI phenotype deposit significantly more mineral in vitro compared to osteoblasts from patients with a severe OI phenotype. RNA-Seq reveals that all OI patients share upregulated proteasomal protein degradation and downregulated protein translation. Furthermore, puromycin assay of neosynthesized proteins shows osteoblasts from both severe patients have increased protein synthesis compared to osteoblasts from moderate patients. Investigating the UPR signaling pathway, we demonstrated that osteoblasts from one moderate patient had different expression patterns of UPR-related proteins compared to osteoblasts from other three OI patients. Specifically, osteoblasts from a patient with moderate OI and a G589S substitution have increased BIP consistent with activation of PERK pathway, as well as decreased PDI, important for collagen folding. In addition, comparing two Brtl mouse models, expressing different substituting residues at the same collagen α1(I) glycine 349, we reveal that the Brtl Ser mouse has a more severe bone phenotype compared to our previously published Brtl Cys mouse, indicating that serine residue at this location has a more deleterious effect on bone than cysteine. In addition, this study reveals that both human and murine OI osteoblasts have elongated mitochondria, confirming in dominant OI the same effect we first reported in *TMEM38B*-deficient osteoblasts causing recessive human OI [15].

### 3.1. Potential Pathways in Phenotypic Variability

We analyzed multiple signaling pathways enriched in the functional enrichment analysis of the RNA-Seq data to reveal which might be involved in the variability of OI phenotype. We showed that the common upregulated pathway all OI patients shared as a group was related to proteasomal protein degradation, while the common downregulated pathway was protein translation. In addition, proteasomal protein degradation and ubiquitination were also shown to be among the most affected pathways when each OI patient was compared separately to matched controls. Previously published work on primary fibroblasts from patients with lethal type II OI reported that pro-α1(I) chains with C-propeptide mutations that impaired triple helix assembly were degraded [16], which suggests that degradation via proteasomes could be a common pathway in dominant OI.

The combination of upregulated protein degradation and downregulated protein translation led us to investigate the synthesis of total cellular proteins by OI osteoblasts. Total protein synthesis was significantly increased in osteoblasts from severe patients from both sets, compared to osteoblasts from moderate patients. Such an increase in total proteins suggests that osteoblasts from patients with severe phenotype produce more protein to compensate for their higher retention of mutant collagen in cells, likely compromising general secretion [17]. Interestingly, the study by Han et al. showed the increased protein synthesis was induced by CHOP (C/EBP homologous protein), an apoptotic factor, that further caused oxidative stress and cell death [18]. We utilized immunohistochemistry to demonstrate increased CHOP-positive cells in OI osteoblasts with a trend toward a further differentiated increase in osteoblasts from patients with a severe vs. moderate phenotype. This suggests that restricting protein synthesis could be a potential mechanistical target for OI therapy. Pairs of OI osteoblasts with same mutation showed similar effects on expression levels of osteoblast marker genes when compared to the control. However, when comparing the phenotypes within each pair, there were inconsistent directions in expression levels.

### 3.2. Hypermineralization in Phenotypic Variability

A common feature of almost all OI types is hypermineralization of bone [19,20], which is the main cause of OI bone brittleness. Exceptions to this rule are types XIV and XV OI, caused by mutations in *TMEM38B* and *WNT1*, respectively, in which osteoblasts showed decreased deposition of minerals by in vitro mineralization assay [21,22]. Our in vitro data showed that osteoblasts from both patients with a severe phenotype deposited significantly less mineral compared to osteoblasts from patients with a moderate phenotype. This was our first indication that in vitro mineralization may be associated with OI phenotypic variability. The decreased in vitro mineralization of OI osteoblasts during a 3-week differentiation is in contrast to the well-established hypermineralization of OI bone tissue detected by BMDD, which does not differ between types III and IV OI [23]. The delayed differentiation of OI osteoblasts, as well as alterations in other pathways related to collagen deposition into the matrix, may be reflected in the in vitro mineralization data.

### 3.3. UPR Signaling in Phenotypic Variability

The UPR signaling pathway is known to be activated in both dominant and recessive forms of OI [7,8]. Studies of patient fibroblasts carrying mutations in *COL1A1* or *COL1A2* revealed upregulated BIP protein levels, a main activator of UPR pathway sensors. One interesting finding was that BIP was expressed differently in cells from patients who had different OI severity while carrying an identical mutation [7]. BIP levels were increased in cells from patients with a lethal phenotype, while in cells from patients with a moderately severe phenotype, the level of BIP was normal. PERK was upregulated in most cases, indicating activation of the PERK branch of UPR pathway. PDI, which catalyzes the formation of disulfide bonds in the ER during protein folding [24], was elevated in OI fibroblasts, except for cells from patients with a lethal phenotype [7]. Another study showed activation of the UPR in fibroblasts with recessive OI types, with mutations in *CRTAP*, *P3H1*, or *PPIB*; however, not all cases showed upregulation of BIP, PERK, and PDI [8]. In addition, iPSC-derived mesenchymal stem cells from fibroblasts of OI patients carrying glycine mutations of *COL1A1* (c.1814G > C) and *COL1A2* (c.1072G > A) showed increased expression of UPR genes leading to increased apoptotic cell death [25].

Our results showed alterations of BIP unrelated to OI severity and mutation position along the collagen type I chain. Only osteoblasts from the moderate patient with a G589S mutation showed upregulation of BIP consistent with PERK activation, while PDI was reduced. On other hand, osteoblasts from the other three OI patients showed downregulation of BIP, while PDI was increased. Reduction in PDI increases the accumulation of misfolded proteins and can cause ER stress, which might explain the activation of the PERK branch. This suggests that, at this juncture, even though the UPR pathway is altered and is an important pathway in OI, we are unable to support the UPR as a major predictor of OI severity. Expanding the investigation to larger sets of factors that might mediate and link the UPR and collagen synthesis and to studies of additional pairs of OI osteoblasts is warranted.

ERO1Lα, which together with PDI facilitates oxidative protein folding, was generally elevated in osteoblasts from OI patients throughout differentiation. Alterations in ERO1Lα were shown to be critical for development of certain conditions such as cancer, diabetes, and neurodegenerative diseases. [9]. In addition, it was shown that prolonged ER stress upregulates ERO1Lα via CHOP expression, which is an apoptotic factor and also a part of UPR, by increasing reactive oxygen species (ROS) and apoptosis [26]. However, some studies speculate that the source of ROS could be mitochondrial [27].

### 3.4. Murine Models for Phenotypic Variability

Our murine models Brtl Ser and Brtl Cys were investigated to understand the phenotypic variability in the OI skeletal phenotype resulting from different glycine substituting residues at the identical collagen glycine. The Brtl Ser mouse is almost invariably perinatal lethal, with few surviving animals, of which the longest survived 9 weeks. Unfortunately, this limited our planned study, as breeding did not yield enough animals for comparison. However, our limited murine study showed clearly that the Brtl Ser mouse bone phenotype is significantly more severe than the Brtl Cys mouse, especially shown by microCT data on trabecular and cortical bone parameters.

One interesting and profound difference was in expression of HSP47, a collagen-specific chaperone, which was highly expressed in Brtl Cys calvarial osteoblasts and co-localized with collagen by immunocytochemistry. On other hand, HSP47 was significantly reduced in Brtl Ser calvarial cells with reduced co-localization with collagen, suggesting a severe impairment of collagen synthesis in Brtl Ser mice. This association of reduced HSP47 with a more severe OI murine phenotype may amplify the results of a recent study in which exogenous rHSP47 was administered to OI type VII and VIII human fibroblasts, as well as to a zebrafish OI type VIII model. HSP47 administration significantly reduced collagen post-translational overmodification and increased collagen secretion and matrix incorporation, associated with significantly improved bone mineralization in vivo. In addition, a mutation-dependent effect was shown in fibroblasts from patients with collagen I mutations, with rHSP47 being effective only in fibroblasts with the most N-terminal defect [28]. Further studies will focus on the mechanism of the diminished HSP47 response and on the skeletal phenotype of Brtl Ser when the mutation is activated in more mature osteoblasts rather than by EIIa-Cre.

### 3.5. Mitochondria Alterations in OI and Phenotypic Variability

Our findings revealed that osteoblasts from both human and murine OI with dominant inheritance showed alterations in mitochondrial morphology on electron microscopy. In addition, RNA-Seq functional enrichment analysis revealed the mitochondrial translation pathway was among enriched pathways in which genes were downregulated, as shown in individual comparison of each OI patient osteoblasts to the control, supporting the electron microscopy data. The severity of the OI phenotype was not a factor in inducing changes in mitochondrial morphology. Rather, mitochondrial dysfunction is a contributing factor to OI pathophysiology. The first evidence of mitochondrial dysfunction was showed in muscle tissue of OI murine models such as oim/oim and Jrt [29,30]. A recent study showed that integrated stress response (ISR), triggered by ER collagen accumulation in osteoblasts from dominant G610C OI mice, was induced and regulated by changes in mitochondrial Hspa9/HSP70 (mt-HSP70) [31]. Our recent study showed for the first time that alterations in mitochondrial function is a contributing factor to pathology of OI osteoblasts. Absence of *TMEM38B*/TRIC-B in type XIV OI osteoblasts caused striking changes in mitochondrial morphology, with an elongated shape, crystolysis, and increased superoxide production [15]. The ER and mitochondria contact sites (ER-MCSs) regulate many mitochondrial functions, including mitochondrial fusion/fission, calcium, and lipid trafficking [32]. Further studies on the mitochondrial role in OI bone pathology, both dominant and recessive, and in OI phenotypic variability will be important to shed light on this potential molecular target for OI therapeutics.

### 3.6. Final Remarks

The results of our study revealed several significant ways in which osteoblast function can play a role in modifying OI phenotype. Mineralization, protein synthesis, and mitochondrial function in OI osteoblasts are among the most prominent modifying factors that were found to correlate with OI phenotypic variability.

However, our study has a few limitations. The comparison of two OI patients per genotype represents the major limitation, as genetic factors in other individuals could affect the phenotype. Although bone samples from different individuals with identical OI genotypes are difficult to obtain, expansion of these studies to a higher sample size by studying genotypically paired individuals with additional OI mutations is warranted to validate and expand these findings. One of the control donors had non-OI skeletal deformities; however, the bone chips were collected from the donor’s healthy proximal femur. For the murine investigation, the mixed CD1-Brtl genetic background in both Brtl murine models, which was established to obtain Brtl Cys survival, could induce greater variability in phenotype. However, the mixed background concurrently provides advantages, one of which is its better suitability to identifying modifying factors.

## 4. Materials and Methods

### 4.1. Generation of Brtl Ser Mice

The Brtl Ser mouse is a conditional knock-in murine model generated by Ozgene Pty Ltd. (Perth, Australia). The introduced mutation results in a glycine-to-serine substitution in exon 23 of murine *Col1a1* locus at glycine 349 (helical Gly349Ser, p.Gly527Ser), which was activated by mating the conditional mice with EIIa-Cre mice (strain background of the WT). The conditional knock-in allele contains a floxed WT cDNA of *Col1a1* exons 23–51. This conditional knock-in mouse line was generated by homologous recombination of a targeting construct into C57BL6 Bruce4 ES cells. Correctly targeted ES cells were injected into host blastocysts to generate chimeric animals. Chimeras were crossed to a flp driver line (conditional) to excise the neomycin cassette and generate heterozygous conditional knock-in mice. The structural diagrams of WT, conditional, and knock-in alleles are shown in Supplemental Figure S1. Due to the lethal OI phenotype that might be induced by presence of a Gly349 substitution in a C57BL6 background, mice with the conditional allele were backcrossed five times with WT mice with CD1-Brtl mixed background. All the animal experiments presented in this study are with CD1-Brtl mixed background. All animal procedures and care were performed under a protocol approved by the local NICHD Animal Care and Use Committee (ACUC), with protocol number NICHD ASP 24-075.

Murine genotypes were determined by PCR amplification of DNA extracted from mouse tail clips using REDExtract-N-Amp™ Tissue PCR Kit (Sigma-Aldrich; St. Louis, MO, USA and the following primers: 5′-GTGTCTCTCCTCATTTGCTCTTAG-3′ Forward and 5′-CCATACAGGACAGAGGATCTTCTC-3′ Reverse for WT and Brtl Ser knock-in allele yielding 545 bp and 608 bp fragments, respectively; 5′-GACCGATGGATTCCCGTTCGAGTACGGAA-3′ Forward and 5′-CCATACAGGACAGAGGATCTTCTCACCTTG-3′ Reverse for the conditional allele, yielding a 961 bp fragment. PCR conditions for WT and Brtl Ser knock-in reaction were as follows: 1 cycle of initial denaturation at 95 °C for 3 min, 35 cycles comprising denaturation at 95 °C for 30 s, annealing at 60 °C for 30 s and elongation at 72 °C for 45 s, and then 1 cycle of the final elongation at 72 °C for 7 min. PCR conditions for conditional reaction were as follows: 1 cycle of initial denaturation at 95 °C for 5 min, 35 cycles comprising denaturation at 95 °C for 45 s, annealing at 61 °C for 45 s and elongation at 72 °C for 1 min 15 s, and then 1 cycle of the final elongation at 72 °C for 7 min.

### 4.2. Human Subjects

The local Institutional Review Board (IRB) of the National Institutes of Health approved protocol 92-CH-0034 on 5 November 1991, which is currently closed and in data analysis for other studies.

Inclusion criteria were individuals aged 3–8 years old with clinical and biochemical criteria of types III or IV whose height is less than the 3rd percentile for age. Participants were required to have radiographic evidence of unfused long bone epiphyses; femur angulation greater than 60 degrees must have surgical correction. Participants must have scoliosis with a curve of less than 40 degrees or stable for the prior 2 years.

Exclusion criteria were as follows: Participants with corrective rods in their spine at enrollment will be excluded. Children will be screened by spiral CT scan with MRI confirmation after enrollment; those with severe BI will be excluded from further participation in this study. Failure to comply with protocol procedures or visits will also be a criterion for withdrawal.

Cells from OI prepubertal patients were derived from iliac crest bone biopsies obtained as part of the protocol diagnostic workup. For the G352S mutation, at the time of biopsy, the type III patient was a 13-year-old female, while the type IV patient was a 13-year-old male. For the G589S mutation, the type III patient was a 5-year-old male, while the type IV patient was a 6-year-old female. Healthy control osteoblasts were obtained from three donors, all prepubertal males, who were 3, 4, and 10 years old at the time of their surgical procedure. The 3-year-old donor showed no skeletal-related abnormalities. The osteoblasts from the 4-year-old donor are a well-established control that has been extensively used in the JCM lab [33]. The 10-year-old donor had skeletal deformities not related to OI; complete information on control osteoblasts from this donor was previously published [15]. Written, informed consent was obtained from all subjects or their respective guardians (JCM for probands and 4-year-old control and MT for 3-year- and 10-year-old controls).

### 4.3. Patient Bone Samples and Murine Calvarial Cells

Primary osteoblasts were derived from surgical discard bone chips of OI patients and healthy donors. Bone chips were minced and processed according to the Robey and Termine protocol [34]. Bone chips were digested with Collagenase P (Roche, Basel, Switzerland) for 2 h at 37 °C in alpha-minimal essential medium (αMEM, Gibco, Grand Island, NY, USA) with Penicillin-Streptomycin antibiotics (100 units/mL Penicillin; 100 µg/mL Streptomycin, Gibco, Grand Island, NY, USA). After digestion, bone chips were transferred to the culture dish with αMEM media supplemented with 1% Penicillin–Streptomycin and 10% fetal bovine serum (Gem Cell, Gemini Bio, West Sacramento, CA, USA) at 37 °C (8% CO_2_) for several weeks until osteoblasts emerged onto the culture dish.

Murine calvarial osteoblasts were isolated from postnatal Day 1 (P1) wildtype, Brtl Ser, and Brtl Cys pups according to the standard protocol for isolating calvarial osteoblasts [35]. Cells were collected from collagenase digestion of calvaria bone and subsequently cultured in αMEM media supplemented with 1% Penicillin–Streptomycin and 10% fetal bovine serum at 37 °C (8% CO_2_) until subconfluency.

### 4.4. Alizarin Red Staining

Control and patient osteoblasts were seeded in 12-well plates in technical triplicates and differentiated for 6 weeks in osteoblast differentiation media (αMEM media, 10% FBS, 1% Penicillin–Streptomycin) supplemented with β-glycerol phosphate disodium salt hydrate (2.5 mM, Sigma-Aldrich; St. Louis, MO, USA), (+)-Sodium L-ascorbate (50 µg/mL, Sigma-Aldrich; St. Louis, MO, USA), dexamethasone (10 nM, Sigma-Aldrich; St. Louis, MO, USA), and recombinant BMP-2 (100 ng/mL, 355-BM, R&D Systems, Minneapolis, MN, USA). WT, Brtl Ser, and Brtl Cys calvarial osteoblasts were treated the same way, except without dexamethasone in the media. At the end point of differentiation, cells were fixed with 4% paraformaldehyde (PFA) for 10 min and then washed with PBS. Fixed cells were stained for 30 min with 1% Alizarin Red/2% ethanol (pH 4.1–4.3) at room temperature. Excess dye was removed with 4 washes with distilled water. Representative stained cultures were photographed. For quantitation, dye was extracted with extraction buffer (0.5 M HCl, 5% SDS) for 10 min at room temperature, and the absorbance was read at 405 nm.

### 4.5. RNA Extraction and Quantitative Real-Time PCR

Technical triplicates of control and patient osteoblasts were harvested on Days 0, 7, 14, and 21 of osteoblast differentiation. RNA was extracted using RNeasy Mini Kit according to manufacturer’s instructions (Qiagen, 74106, Germantown, MD, USA). RNA concentration was measured by NanoDrop spectrophotometer.

cDNA was synthesized using a high-capacity cDNA reverse transcription kit (Applied Biosystems, Foster City, CA, USA). For each RT-qPCR reaction, 20 ng of cDNA was used. RT-qPCR reaction was carried out on Quant Studio 6 Flex (Applied Biosystems, Foster City, CA, USA) using TaqMan fast universal PCR master mix. mRNA expression levels of genes were normalized to *B2M* reference gene based on comparative Ct method (^ΔΔ^Ct). The TaqMan probes sequences are as follows: *SP7* (Hs01866874_s1), *RUNX2* (Hs01047973_m1), *COL1A1* (Hs00164004_m1), *ALPL* (Hs01029144_m1), *IBSP* (Hs00173720_m1), *B2M* (Hs00984230_m1) (Applied Biosystems, Foster City, CA, USA).

### 4.6. Whole-Mount Skeletal Staining and X-Ray Imaging

Whole-mount skeletal staining of WT and Brtl Ser pups at postnatal day 1 was performed according to the protocol of Rigueur and Lyons [36], using Alcian Blue and Alizarin Red staining (Sigma-Aldrich, St. Louis, MO, USA). First, pups were scalded in 65 °C hot water for 20–30 s to facilitate the skin removal and the permeabilization of tissues. Skin, eyes, internal organs, and adipose tissue were removed. Then, samples were fixed in 95% ethanol overnight, followed by acetone fixation overnight. Samples were stained for cartilage by submerging them in Alcian Blue staining overnight. De-staining was performed by washing the samples with two changes of 70% ethanol and with 95% ethanol overnight. The next day, samples were pre-cleared with 1% KOH for 1 h, followed by Alizarin Red staining for 4 h to stain bone tissue. Then, excess red dye was removed by submerging samples in 50% glycerol/50% (1%) KOH until tissue appeared transparent. For long-term storage, samples were transferred to 100% glycerol.

X-ray imaging of WT and Brtl Ser postnatal P1 old pups or adult mice were performed by using an UltraFocus^DXA^ (Hologic Life Sciences-Faxitron^®^, Marlborough, MA, USA).

### 4.7. Micro-Computed Tomography

Femurs from WT and Brtl Ser mice were subject to µCT measurement of trabecular and cortical bone parameters. Femora were scanned in water using a SkyScan 1176 (Bruker, Billerica, MA, USA) at 9 µm isotropic voxel size, with a 0.3° rotation angle; 2 frames were averaged and filtered by 0.5 mm aluminum. A source voltage of 50 kVp and a current of 500 µA were used. Images were reconstructed and calibrated using manufacturer supplied phantoms. Femoral cortical regions of interest spanned 15% of total bone length (centered between the proximal end of the distal femoral growth plate and distal end of the lateral third trochanter). Trabecular regions spanned 10% of total bone length extending proximally from the proximal end of the distal femoral growth plate. Otsu thresholding was performed to segment bone from non-bone voxels and manufacturer-provided software (version 5.40, CTAnalysis, Bruker, Billerica, MA, USA) was used to generate results.

### 4.8. Biochemical Analysis of Type I Collagen

Steady-state collagen analysis was performed as previously described [37]. Control and patient osteoblasts, as well as wildtype, Brtl Cys, and Brtl Ser murine calvarial osteoblasts, were seeded in 6-well plates. Subconfluent cells were treated with collagen stimulation media (αMEM, 1% Penicillin–Streptomycin antibiotics, 50 µg/mL ascorbic acid) for 2 h, followed by incubation with L-[2,3,4,5-^3^H] proline (103 Ci/mmol) for 18 h. After collection, both media and cell samples were centrifuged at 10,000 rpm for 20 min at 4 °C to pellet debris; then, collagen was precipitated at 4 °C with 176 mg/mL ammonium sulfate for 3 h. Collagens were pelleted by centrifugation at 37,000× *g* and resuspended in 0.2 M NaCl, 0.05 M Tris, pH 7.5. Finally, collagens were digested with pepsin (50 μg/mL) for 4 h, precipitated with ammonium bicarbonate, followed by separation of collagen alpha chains on 5% SDS-Urea–Polyacrylamide gels, and subsequently visualized by autoradiography.

Amino acid analysis of hydroxylysine, lysine, hydroxyproline, and proline content of secreted type I collagen from control and patient osteoblasts was performed by high-pressure liquid chromatography on a Hitachi L8900 Amino Acid Analyzer (AAA Service Lab, Damascus, OR, USA).

### 4.9. Collagen Secretion

For absolute quantification of collagen secretion, technical triplicates of WT and Brtl Ser calvarial osteoblasts were seeded in 6-well plates, allowed to grow until confluent, and then incubated overnight in αMEM media without FBS. The following day, media from each sample were collected, supplemented with protease inhibitor cocktail (P8340, Sigma-Aldrich; St. Louis, MO, USA), and concentrated using Amicon Ultra-4 centrifugal filter (Millipore, Billerica, MA, USA) at 3300 rpms for 1 h. After media collection, each sample was trypsinized and cells were counted. Secreted collagen was quantified using Sircol Soluble Collagen Assay (Biocolor Ltd., Northern Ireland, UK) according to the manufacturer’s instructions.

### 4.10. Collagen Folding Assay

The collagen folding assay was performed as previously described [38]. WT and Brtl Ser osteoblasts were cultured in αMEM media with 50 µg/mL ascorbic acid overnight and then incubated in serum free media containing 50 µg/mL ascorbic acid for 2 h. Cells were pulsed for 15 min with 1 µCi/well ^14^C-proline, followed by collection of cell lysates every 5 min. Lysates were digested in 0.2% Triton X-100/PBS containing 100 µg/mL trypsin and 250 µg/mL chymotrypsin. Soybean trypsin inhibitor was added to stop the digestion. Samples were precipitated overnight, collected by centrifugation, and electrophoresed on NuPAGE 3–8% tris-acetate gels. Collagen chains were quantitated by densitometry and percentage of folded chains was calculated.

### 4.11. Transmission Electron Microscopy of Murine Dermal Collagen Fibrils

Dermal punch biopsies of abdominal skin were obtained from WT and Brtl Ser mice. Following 24 h fixation in 2.5% glutaraldehyde, the samples were further processed as previously described [39]. Following fixation, samples were treated with 1% osmium tetroxide followed by en bloc staining with 2% uranyl acetate. Then, samples were dehydrated and infiltrated with Spurr’s plastic resin. Sections with a thickness of 600–800-Å were obtained with an AO Reichert Ultracut ultramicrotome mounted on copper grids and then stained with lead citrate. The stained grids were inspected with a Zeiss EM10 CA transmission electron microscope (Zeiss, Oberkochen, Germany), and representative areas were photographed. Diameters of approximately 200 fibrils of each dermal biopsy were measured from four images, and the average was presented.

### 4.12. Immunocytochemistry

Technical triplicates of control and patient osteoblasts were seeded in 8-well glass chamber slides. After 2 days, cells were treated with Tunicamycin for 8 h. Cells were fixed with 4% PFA for 15 min followed by PBS washes 3 times for 5 min. Blocking was performed for 1 h in buffer containing 5% normal goat serum (Jackson Immuno Research) and 0.1% Triton in PBS. Incubation with the primary antibody was for 2 h at room temperature (CHOP (2895) 1:400; LC3A/B (4108) 1:400, Cell Signaling, Danvers, MA, USA). After PBS washes, cells were incubated with secondary antibodies (Alexa Fluor™ 555 donkey anti-mouse IgG, 1:200; Alexa Fluor™ 488 goat anti-rabbit IgG, 1:200; Invitrogen, Waltham, CA, USA) for 1 h. Cells were washed with PBS and counterstained with DAPI (1 μg/mL) for 10 min. Slides were processed with Vectashield^®^ antifade mounting medium (H-1000, Vector Laboratories, Burlingame, CA, USA) and sealed with cover slips. Images were taken with a Zeiss LSM 710 Confocal Microscope (Zeiss, Oberkochen, Germany). The same protocol was used for immunocytochemistry staining of WT, Brtl Ser, and Brtl Cys calvarial osteoblasts, but without Tunicamycin treatment. Primary antibodies used were HSP47 (ADI-SPA-470) 1:500, Enzo Life Sciences, Farmingdale, NY, USA; COL1A1 [LF-68, a generous gift from Dr. Larry Fisher, NIH] [40] 1:1000; Phalloidin (A12379) 1:1000, Invitrogen, Waltham, CA, USA.

### 4.13. Puromycin Assay

Technical triplicates of control and patient osteoblasts were seeded in 10 cm plates and grown until subconfluent. Cells were cultured in αMEM media with 1% Penicillin–Streptomycin antibiotics without FBS for 16 h. The following day, cells were pulsed with 1 μM puromycin (P8833, Sigma-Aldrich; St. Louis, MO, USA) for 1 h, except for control plates that were used as a negative (no puromycin) control. Cells were harvested, pelleted, washed with PBS, flash-frozen, and stored at −20 °C until use. Cell pellets were lysed by sonication in RIPA buffer containing protease inhibitor cocktail (P8340, Sigma-Aldrich; St. Louis, MO, USA) and centrifuged at 14,000× *g* for 15 min. Samples were subjected to Western blot using mouse anti-Puromycin antibody (1:5,000; MABE343, Sigma-Aldrich; St. Louis, MO, USA).

### 4.14. Western Blot

Technical duplicates of control and patient osteoblasts, as well as WT, Brtl Ser, and Brtl Cys murine calvarial osteoblasts, were seeded in 6 cm plates in triplicate for each timepoint and harvested on Days 0, 7, 14, and 21 of osteoblast differentiation in RIPA buffer containing protease inhibitor cocktail (P8340, Sigma-Aldrich; St. Louis, MO, USA). Samples were further treated and Western blot was performed as previously described [15]. Dilutions of primary antibodies are as follows: PERK (5683) 1:1000; IRE1α (3294) 1:1000; BIP (3177) 1:1000; PDI (3501) 1:1000; Ero1-Lα (3264) 1:1000; GAPDH (D4C6R) 1:1000; GAPDH (D16H11, XP(R)) 1:1000—all from Cell Signaling, Danvers, MA, USA; HSP47 (ADI-SPA-470) 1:1000—Enzo Life Sciences, Farmingdale, NY, USA. The membrane was developed using LI-COR Odyssey^®^ CLx Western Blot imager (Lincoln, NE, USA).

### 4.15. RNA Sequencing (RNA-Seq) and Data Analysis

RNA from technical triplicates of control and patient osteoblasts on Days 0, 7, 14, and 21 of osteoblast differentiation, previously extracted with an RNeasy Mini Kit, were subject to bulk RNA-Seq sequencing. Total RNA samples (~2 µg) were purified with Poly-A extraction and then used to construct RNA-Seq libraries with specific barcodes using the Illumina TruSeq Stranded mRNA Library Prep Kit. All barcoded RNA-Seq libraries were pooled together and sequenced using an Illumina HiSeq 2500 sequencer with four technical replicates for each sample to generate 7–50 million 2 × 100 bp reads for each sample. The raw data were demultiplexed and analyzed further using lcdb-wf v1.4.1rc https://lcdb.github.io/lcdb-wf/ (accessed on 14 June 2019) according to the following steps: Raw sequence reads were trimmed with cutadapt v2.3 [41] to remove any adapters while performing light quality trimming with parameters ‘-a AGATCGGAAGAGC-A AGATCGGAAGAGC-q 20 –minimum-length 25.’ Sequencing library quality was assessed with fastqc v0.11.8 with default parameters. The presence of common sequencing contaminants was evaluated with fastq-screen v0.13.0 with parameters ‘–subset 100,000 –aligner bowtie2.’ Trimmed reads were mapped to the Homo sapiens reference genome (GENCODE v28) using HISAT2 v2.1.0 [42] in paired-end mode with parameters ‘–no-unal’. Uniquely aligned reads mapping to genes were quantified using the featureCounts program of the subread package v1.6.4 [43] using the Homo sapiens reference (GENCODE v28) annotations. Differential expression was performed using raw counts supplied to DESeq2 v1.22.1 [44]. A gene was considered differentially expressed if the false discovery rate (FDR) was <0.1. Functional enrichment was performed for GO Biological Process, Cellular Component and Molecular Function using the ClusterProfiler v 3.10.1 [45] function enrichGO.

### 4.16. Electron Microscopy of Cells

Control and patient osteoblasts, as well as WT, Brtl Ser, and Brtl Cys calvarial osteoblasts, were seeded in 6-well plates and were further processed for electron microscopy, as previously described [15]. At the subconfluent level, cells were fixed with EM fixative solution (2% glutaraldehyde in 0.1 M cacodylate buffer, pH 7.4) for 1 h at room temperature followed by overnight fixation at 4 °C. EM fixative was replaced with PBS and the cell plates were processed and embedded at room temperature in a fume hood. Following fixation, the cells were washed 2 times for 10 min in cacodylate buffer before post-fixation of 1 h in osmium tetroxide (1% *v*/*v*). The cells were then washed 2 times with water and 1 time with acetate buffer (0.1 M, pH 4.5) before en bloc stain in 0.5% *w*/*v* uranyl acetate (0.5% *v*/*v*) in acetate buffer (0.1 M, pH 4.5) for 1 h. The cells were dehydrated with multiple washes of EM grade ethanol at 35%, 50%, 75%, 95%, and 100%, consecutively. The cells were then washed with pure epoxy resin (Poly/Bed^®^ 812, Polysciences, Inc., Warrington, PA, USA) overnight. Then, the cells were washed with pure resin prior to embedding, after which the cell plates were placed in a 55 °C oven to cure for 48 h. The resin blocks were separated from the plate and examined under an inverted microscope to select an area with considerable number of cells. The preferred area was removed from the block, trimmed, and thinly sectioned using an ultramicrotome equipped with a diamond knife. The thin sections were mounted onto copper mesh grids for counter staining with uranyl acetate and lead citrate. The grids were then carbon-coated in a vacuum evaporator. After that, the grids were ready to be scanned and imaged with a Hitachi Electron microscope (H7650), which operated at 80 kv, with a CCD camera to capture digital images.

### 4.17. Statistics

For statistical analysis, a 2-tailed Student’s *t* test was utilized. Data are presented as means ± SEM. The value of *p* < 0.05 was considered significant.

## Figures and Tables

**Table 1:** Comparison of microCT cortical and trabecular bone parameters between 7-week-old Brtl Ser and 6-week-old Brtl Cys mice.

Cortical ROI	WT	Brtl Ser	* WT	* Brtl Cys
Total Cross-Sectional Area (mm^2^)	2.70	1.58	1.1	0.75
Cross-Sectional Bone Area (mm^2^)	0.93	0.44	N/A	N/A
Bending Moment of Inertia (lly) (mm^4^)	0.23	0.07	0.23	0.13
Cortical Thickness (mm)	0.15	0.10	0.25	0.18
Marrow Area (mm^2^)	1.78	1.15	N/A	N/A
** Trabecular ROI**	** WT**	** Brtl Ser**	* WT	* Brtl Cys
BV/TV (%)	27.60	3.59	16	10
Tb.N (1/mm)	4.62	0.75	5.0	3.3
Tb.Th (mm)	0.06	0.05	0.031	0.030
Tb.Sp (mm)	0.13	0.50	N/A	N/A

* values previously reported by Uveges et al. [12]. N/A—data are not available.

## Data Availability

Data will be made available on request. Full electronic RNA-Seq data will be available in the database of Genotypes and Phenotypes (dbGaP). Data is contained within the article or Supplementary Materials.

## Supplementary Materials

The following supporting information can be downloaded at https://www.mdpi.com/article/10.3390/ijms262311722/s1.
