# Supplementary material for: The Role of Osteoblasts in Phenotypic Variability of Dominant Osteogenesis Imperfecta: Evidence from Patients and Murine Models
Source: Int J Mol Sci. 2025 Dec 3;26(23):11722. doi: 10.3390/ijms262311722 (PMC12692505; doi:10.3390/ijms262311722)
Supplement: Supplementary file 1 [file ijms-26-11722-s001.zip › Supplemental Figure 1.pdf]

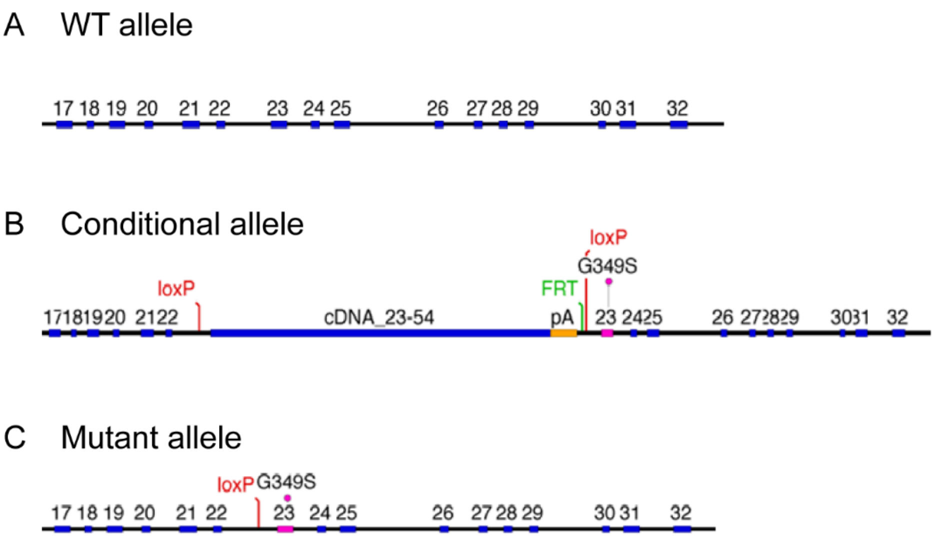

Supplemental Figure S1. The sequence diagrams of A) WT, B) conditional and C) mutant allele of Brtl Ser mouse.
