# Supplementary material for: The Role of Osteoblasts in Phenotypic Variability of Dominant Osteogenesis Imperfecta: Evidence from Patients and Murine Models
Source: Int J Mol Sci. 2025 Dec 3;26(23):11722. doi: 10.3390/ijms262311722 (PMC12692505; doi:10.3390/ijms262311722)
Supplement: Supplementary file 1 [file ijms-26-11722-s001.zip › Supplementary Clinical Information.pdf]

Supplementary Clinical Information

Clinical Case Reports and Radiographs

Patient with a severe phenotypic outcome from a *COL1A1* mutation causing a helical Gly352Ser substitution (**Subject 1**) is a currently 41-year-old Caucasian female. She was born full-term from an uncomplicated pregnancy through Cesarian section due to breech presentation. At delivery, she was noted to have blue sclerae and numerous *in-utero* fractures of femora, rib, clavicle, and skull. This led to a clinical diagnosis of Osteogenesis Imperfecta (OI) prior to discharge 10 days later, later refined by clinical phenotyping to OI Type III. She started sitting independently by age 6 months. At 29 months she was pulling to standing but needed gait aids for cruising, and by 31 months could take many sequential steps with walker and leg braces. Able to walk independently at age 4-5 years. Other developmental milestones were age-appropriate, for sentences at age 2, fine motor hand control at age 3, toilet-trained by 2.5 years.

She was first seen at the NICHD OI clinic at age 2 years and followed through age 22 years. During that time, she had further long bones fractures at her right tibia and left humerus at age 5 years, three bilateral femur fractures within her fourteenth year, and a toe fracture at age 15. By age 17 she had bilateral tibial rods that had been replaced once and bilateral femur rods with the right removed and the left replaced once. She was independently ambulatory with lofstrand forearm crutches or a walker, but most often used a wheelchair. At age 16 years, her plain films showed a marked thoracic spine scoliosis with rib cage and pelvic deformities. At that same visit her lumbar spine DEXA bone mineral density (BMD) was 0.574 gm/cm2, with a Z-score of -3.97. At age 17 years, her length was 105.7cm, which is 50th percentile for 4.25-year-old females per CDC Growth Charts, with BMI of 28.8. Her head circumference was between 50th to 75th percentile for age throughout childhood, measuring 49cm at age 32 months, for example. She received subcutaneous growth hormone in an NICHD protocol for one year from ages 9 to 10 years but never received bisphosphonates.

She has dentinogenesis imperfecta (DI) noted since age 2 years, requiring capping at age 3 years, and later developed generalized gingivitis and multiple caries, but her dental status improved with permanent teeth. She also has basilar invagination (BI), with neurology noting a flattened occiput as early as 3 years. BI was stable through age 17, but incremental progression was noted at age 22 leading to platybasia and Chiari I-type malformation without neurological symptoms other than chronic migraine headaches. Annual audiologic evaluations revealed a mild bilateral conductive hearing loss. No cardiac abnormalities noted on echocardiogram at age 16 years. She has a history of mild asthma controlled with as-needed inhalers, without formal pulmonary function test evaluation.

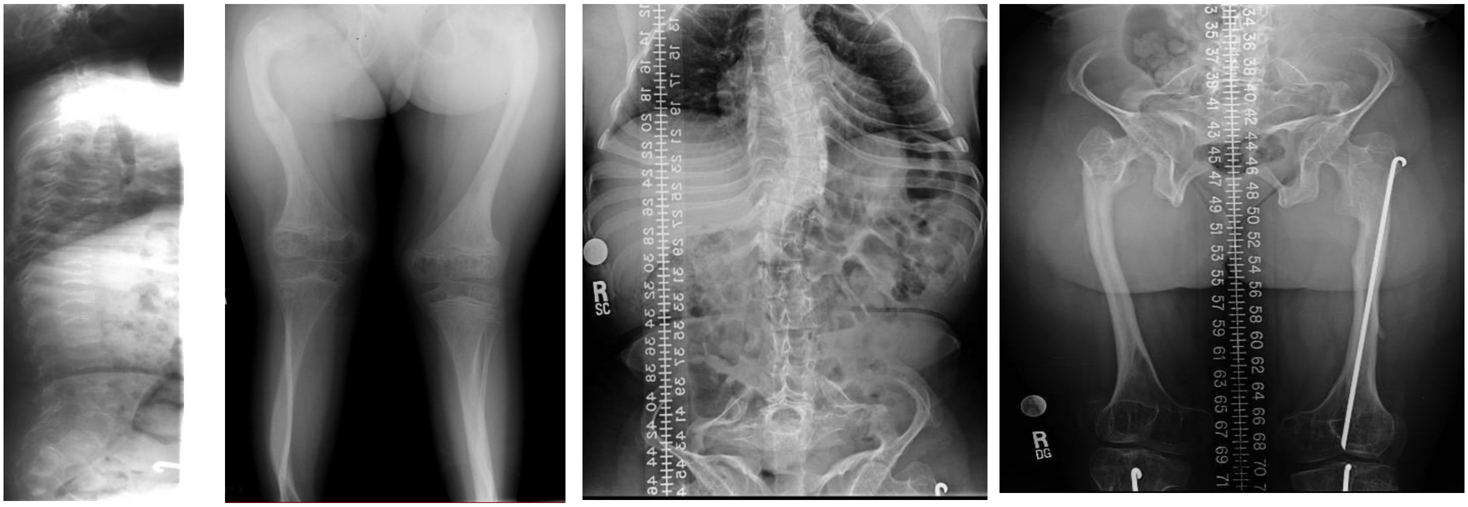

8 years 8 years 18 years 19 years

A) Radiographs of Subject 1 – Gly352Ser type III OI

Patient with a moderate phenotypic outcome from a *COL1A1* mutation causing a helical Gly352Ser substitution (**Subject 2)** is a currently 42-year-old Caucasian male. He was born at 32 weeks estimated gestational age by Cesarian section for *in-utero* suspicion of OI, in a pregnancy complicated by gestational diabetes. His father had severe Type III OI, while both this individual and his older brother had strikingly milder OI than their parent and were diagnosed with Type IV. His prenatal ultrasound revealed bone demineralization, fractures, and lower extremity deformities. He required respiratory support for one week after birth with a transient heart murmur. His sclera had a blue hue. He was followed at this clinic from ages 4-13, then returned at 35-36 years.

His motor development was only mildly delayed. He crawled at age 9 months, walked by 18 months, but only ambulated consistently for about one year around age 6 due to frequent fractures, even with bilateral femur rods both placed at age 3. At initial NIH CC assessment, we noted concerns with coordination and fine motor skills. He had a speech articulation defect, due to a left-sided palate excursion, though language skills were within normal limits. He had approximately 26 fractures between the ages of 2.5-4.5 years old, then 2 right humerus fractures, eight femur fractures, and >10 tibial fractures by age 19 years. By age 19 he had bilateral femur rods (right replaced 3 times, left twice) and bilateral tibial rods. At age 13 years he was independent for transfers after recent fractures, although he used a wheelchair primarily for mobility continuing into adulthood.

At age 13 years his spine had diffuse bony osteopenia with moderate thoracic scoliosis (approximately 24 degrees) and moderate compression of multiple vertebral bodies, His

lumbar spine DXA showed BMD of 0.403 gm/cm2 with Z-score of -4.65. His length at 6.5 years was 102.86cm (50th percentile for 4.2 years) while his head circumference was 51cm (55th percentile for chronological age). His length at age 13 years was 137.8 cm, which was 50th percentile for 9.75 years per CDC growth curves. He received subcutaneous growth hormone in an NICHD protocol at ages 9-10 years; he did not enroll in a bisphosphonate protocol.

He had DI with capping of multiple primary teeth, with DI persisting in permanent teeth at age 36 and mandibular mid-incisors dentures. No BI was seen on monitor imaging through age 13 years, with mild platybasia noted at age 35. Ahe has no significant hearing or cardiac defects through age 36. Pulmonary function tests at ages 11 and 35 showed normal flows, although nighttime CPAP use was required for obstructive sleep apnea by age 35 years.

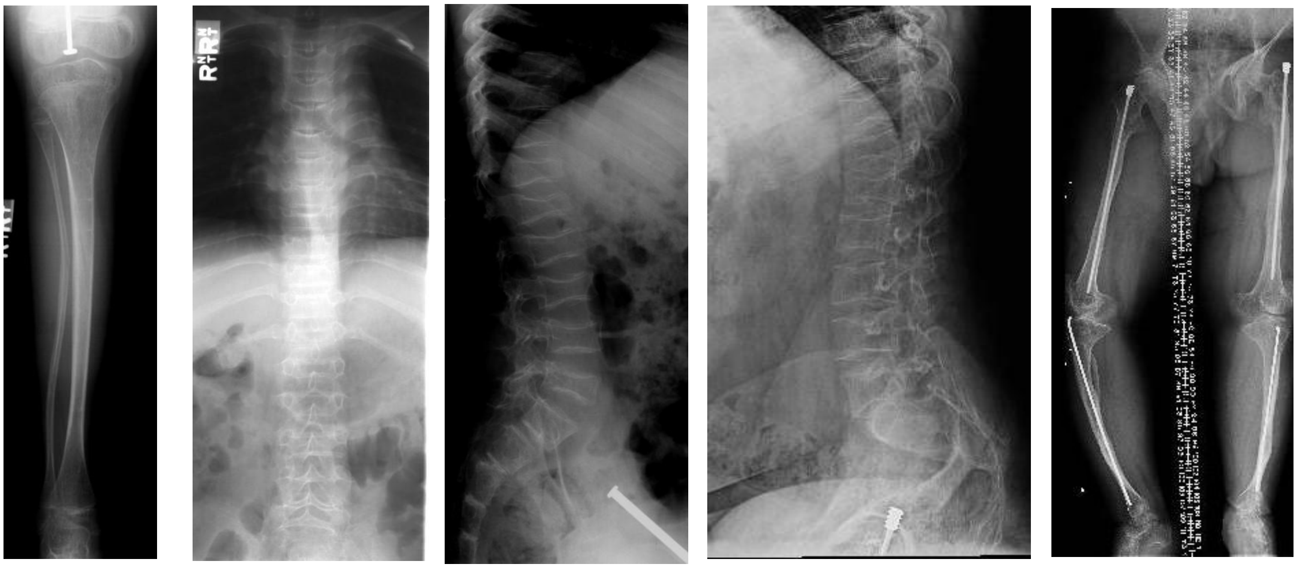

10 years   10 years   10 years   36 years   36 years

B) Radiographs of Subject 2 – Gly352Ser type IV OI

Patient with a severe phenotypic outcome from a *COL1A1* mutation causing a helical Gly589Ser substitution (**Subject 3)** is a currently 33-year-old Caucasian male born at 33 weeks estimated gestational age via Cesarian section as a twin birth (with an unaffected twin sister). In nursery, he was noted to have bilateral femurs fractures, as well as fractures of ribs, left tibia/fibula and left wrist, large fontanels and wide sutures, bowed lower extremities, and blue sclera leading to OI diagnosis. He was discharged after 8 days after receiving ampicillin/gentamycin for positive streptococcal B maternal cervical

culture. Within the first month of life he had multiple additional fractures, including bilateral hips, right femur, and left humerus.

He was followed at the NIH Clinical Center from age 3 months to 30 years. Head control was established by 6 months, sitting unsupported by 9 months, and crawling at 10-12 months. He pulled to stand between 14-15 months and was able to walk at 18 months with leg braces. He had at least one long bone fracture per year in an arm or leg, often more, through age 11, and was considered to have Type III OI. He had more than 30 long bone fractures before age 20. Because of frequent fractures, he had intramedullary rods in bilateral femurs, bilateral tibiae, and left humerus, with multiple replacements in lower extremity rods. As a teenager he was independent for sitting transfers but used a manual wheelchair consistently, transitioning to a motorized chair by age 28.

His spine at age 17 showed marked osteopenia with multiple compression fractures throughout the thoracic spine, worse than the lumbar portion. At 18 years, his lumbar spine DXA yielded a BMD of 0.483 gm/cm2 and Z-score of -5.2. At 3 years his head circumference was 48.5cm (5th percentile for his age) while his length was 72.44cm (50th percentile for 9.25 months). His length at 18 years was 125.4 cm, matching the 50th percentile for age 7.5 years, with a BMI of 48.6. He had received subcutaneous growth hormone from age 6 to approximately 13 years, as well as 11 cycles of pamidronate infusions every 6 months from ages 8 to 11 and an additional 3-day dose once at age 17 years. He completed a one-year setrusumab trial starting at age 26.

He has severe DI, first noted at 17 months, with crowned anterior teeth by age 8, and multiple tooth extractions after fracture by 28 with severe DI of remaining teeth. Marked BI was diagnosed in childhood with occluded foramen magnum and kinking of brainstem at pontomedullary junction seen by age 16 and remained stable through age 28, though progression was measured at 5mm more of the odontoid process above Chamberlain's line from age 13 to 28, with increasing platybasia, although without signs or symptoms of neurological compromise. Audiology exam confirmed a mild right conductive hearing loss by age 16 that was stable through 28 years. Echocardiograms did not show any significant cardiac abnormalities. Pulmonary function deteriorated from a mild restrictive pattern and mild diffusion defect at age 17 to a severe restrictive ventilatory defect by age 29.

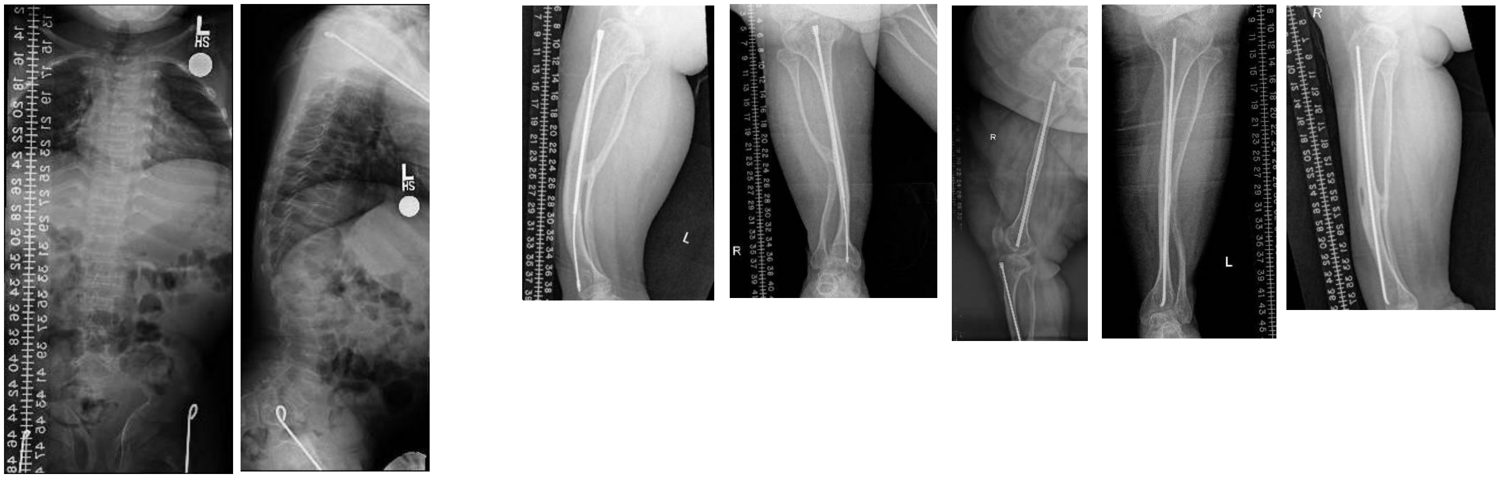

9 years 30 years

C) Radiographs of Subject 3 – Gly589Ser type III OI

Patient with a moderate phenotypic outcome from a *COL1A1* mutation causing a helical Gly589Ser substitution (**Subject 4**) was an African-American female who died at age 30 years. She was born full term via vaginal delivery after an uncomplicated pregnancy and was noted to have leg deformities on delivery and was clinically diagnosed with OI before discharge 2.5 days later. She returned to the hospital at age 13 days for irritability and xrays revealed seven fractures of long bones in various healing stages and marked diffuse osteopenia. She was diagnosed with Type IV.

By 22 months, she was able to walk with assistance of walker or railings. Otherwise, her development was normal. She was seen at the NIH Clinical Center from age 10 months through 17 years. By age 17 years she had several dozen fractures, multiple reported each year, including multiple long bones, scapulae, ribs. In the three years between ages 14-17 she had bilateral femoral neck fractures requiring pinning, right ulna fracture also requiring pinning, bilateral scapulae fractures, 5 left tibial fractures/recurrent fractures. In addition, she had bilateral femoral, tibial, and humeral intramedullary rods. Spinal fusion hardware from T3 to L4 was placed for her kyphosis of approximately 50 degrees and scoliosis of 62 degrees with significant rotation and lordosis. She was independent for transfers and used a posterior rollator walker for home, and a wheelchair in school and for longer distances.

Despite her spinal hardware, she still has a significant high thoracic curve to the right. Her radius DXA at age 17 had a BMD of 0.612 gm/cm2, and T-score of -1.4. Her length was 109.5cm, which matches the 50th percentile for a 5-year-old, with BMI of 25.3. Her head circumference at age 3 of 46.6cm was in the 10th percentile for her age, while her length at 76.6cm then was the 50th percentile for a 14-month-old. She received

intravenous pamidronate therapy at an outside institution every 3 months from age 10 – 17 years.

She had DI of her primary teeth, though none were capped, and significantly decreased pulp chambers were noted. A permanent brace was placed on her lower teeth to stabilize a fractured tooth #24. She was diagnosed with BI by age 5 years, and her BI and platybasia remained relatively stable through age 17 without neurological compromise other than frequent frontal/sinus headaches. Annual audiology exams did not show significant hearing abnormalities, and echocardiograms also did not show cardiac problems other than persistent sinus tachycardia. She had asthma along with significantly restrictive lung disease that required BIPAP at night since she was at least 12 years old through age 17.

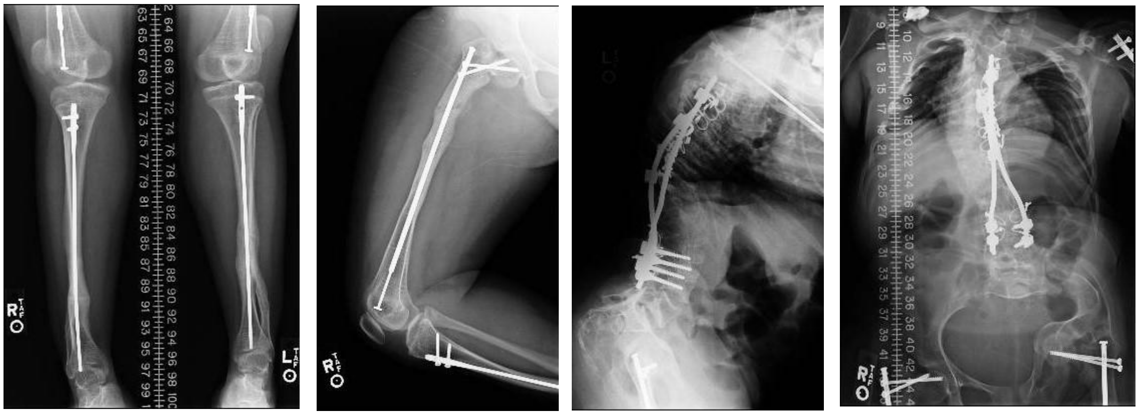

All images - 7 years

D) Radiographs of Subject 4 – Gly589Ser type IV OI
